# From tumor microenvironment to ocular hypertension: unraveling the pathogenesis and therapeutic strategies of cancer-related glaucoma

**DOI:** 10.3389/fmed.2025.1628325

**Published:** 2025-08-14

**Authors:** Bin Lin, Dong-kan Li

**Affiliations:** ^1^Xiamen Eye Center and Eye Institute of Xiamen University, School of Medicine, Xiamen, China; ^2^Xiamen Clinical Research Center for Eye Diseases, Xiamen, Fujian, China; ^3^Xiamen Key Laboratory of Ophthalmology, Xiamen, Fujian, China; ^4^Fujian Key Laboratory of Corneal and Ocular Surface Diseases, Xiamen, Fujian, China; ^5^Xiamen Key Laboratory of Corneal and Ocular Surface Diseases, Xiamen, Fujian, China; ^6^Translational Medicine Institute of Xiamen Eye Center of Xiamen University, Xiamen, Fujian, China

**Keywords:** cancer-related glaucoma, pathogenesis, clinical manifestation, treatment strategies, multidisciplinary collaboration

## Abstract

Cancer is a global health threat, and its incidence and mortality are increasing annually. Cancer-related glaucoma, a severe complication caused by primary or metastatic tumors and their treatments, has complex pathogenic mechanisms. This review aims to clarify the risk factors, classification, diagnosis, and treatment progress of this glaucoma type. Mechanisms include mechanical obstruction, secondary angle closure, neovascularization, inflammation and cytokine release, alterations in aqueous humor dynamics, and secondary hemosiderosis. Clinical manifestations are diverse, such as rapid intraocular pressure increase, neovascular changes, and tumor-related characteristic signs. Treatment requires multidisciplinary cooperation, with primary tumor control as the core, combined with drugs like anti-VEGF agents and targeted therapies, and modified surgeries. Future research should focus on personalized therapeutic strategies, gene therapy applications, integration of multimodal imaging, and optimization of AI models to optimize early intervention and reduce the risk of irreversible optic nerve damage.

## 1 Introduction

Cancer is one of the diseases that seriously threatens human health worldwide, with its incidence and mortality rates increasing year by year. According to data from the World Health Organization (WHO), in 2020, there were over 19 million new cancer cases globally, and nearly 10 million deaths. Ocular and central nervous system tumors, though rare, cause visual dysfunction and systemic complications that severely affect quality of life (1). Advances in cancer diagnosis and treatment have prolonged patient survival, but both tumors and their therapies (such as radiotherapy, chemotherapy, targeted therapy, etc.) can induce ocular complications. Secondary glaucoma, in particular, is concerning due to its risk of irreversible optic nerve damage (2).

Tumors can lead to the development of glaucoma through multiple mechanisms. For example, ocular tumors (such as iris melanoma and retinoblastoma) can directly invade the angle structure or induce neovascular glaucoma (NVG). NVG is one of the most common secondary glaucomas, accounting for 44.9% of cases, with some of those arising from tumors (3, 4). Epidemiological studies show that among pediatric glaucoma cases, secondary glaucoma accounts for as high as 53.4%, and 28% of them are associated with acquired systemic diseases (5, 6). In addition, tumor treatment drugs (such as corticosteroids and immune checkpoint inhibitors) may also cause secondary glaucoma by increasing intraocular pressure or inducing uveitis (7, 8), highlighting the complexity of the relationship between tumors and glaucoma.

With the development of comprehensive cancer treatment, the clinical identification and management of glaucoma secondary to tumors have gradually become an important topic for multidisciplinary collaboration. However, currently, there is a lack of systematic reviews on the epidemiological characteristics, pathological mechanisms, and treatment strategies of such glaucoma (9, 10). Some studies have pointed out that tumor-related glaucoma is often misdiagnosed due to its atypical clinical manifestations. Therefore, we conducted a rigorous review, integrated existing evidence, clarified the risk factors, classification, and progress in diagnosis and treatment of glaucoma secondary to tumors, to provide a reference for clinical practice and call for strengthened ophthalmic follow-up of cancer patients to reduce the risk of irreversible vision loss (2, 11).

## 2 Methods

To comprehensively synthesize the latest advancements in cancer-related glaucoma, we employed a hybrid approach that combines systematic literature retrieval with flexible expansion to ensure both rigor and innovation.

### 2.1 Literature retrieval strategy

A structured Boolean search was initially conducted across major databases (PubMed, Web of Science, Cochrane Library, and Embase) to identify foundational literature. The core search terms were:

(“cancer-related glaucoma” OR “tumor-associated glaucoma” OR “neoplastic glaucoma” OR “secondary glaucoma AND cancer”) AND (mechanism OR pathogenesis OR “clinical manifestation” OR “treatment strategy” OR “neovascularization” OR “intraocular pressure” OR “uveal melanoma” OR “retinoblastoma”) AND [“2020/01/01” (PDAT): “2025/05/01” (PDAT)].

### 2.2 Inclusion and exclusion criteria

#### 2.2.1 Inclusion criteria

Study types: original research (clinical studies, basic experiments), systematic reviews, and meta-analyses.

Content: studies focusing on pathogenesis, clinical manifestations, diagnostic methods, or therapeutic strategies of cancer-related glaucoma; those involving core mechanisms such as the HIF-1α/VEGF pathway and ferroptosis, or emerging technologies including AI-assisted diagnosis and gene editing, as cited in this review.

Publication time: January 2020 to July 2025.

#### 2.2.2 Exclusion criteria

Study types: letters, editorials, and case reports (except for milestone rare cases).

Language: non-English language articles.

Research focus: primary glaucoma or secondary glaucoma unrelated to cancer, such as diabetic glaucoma.

Relevance: literature not explicitly addressing the “cancer-glaucoma” association, merely discussing cancer or glaucoma independently.

Duplication: duplicate publications or studies with overlapping data (the latest or larger-sample studies were retained).

Quality: low-quality literature, including those without clear methodology or statistical analysis.

### 2.3 Literature synthesis approach

After an in-depth review of the retrieved literature, supplementary searches were conducted to incorporate relevant studies that enhance structural completeness, hierarchical richness, and content diversity. This non-traditional approach breaks the “retrieve-then-integrate” framework, prioritizing the divergence of mechanistic logic and inclusiveness of cutting-edge research to better reflect the cross-disciplinary advancements in this field.

### 2.4 Pathogenesis

**FIGURE 1:**
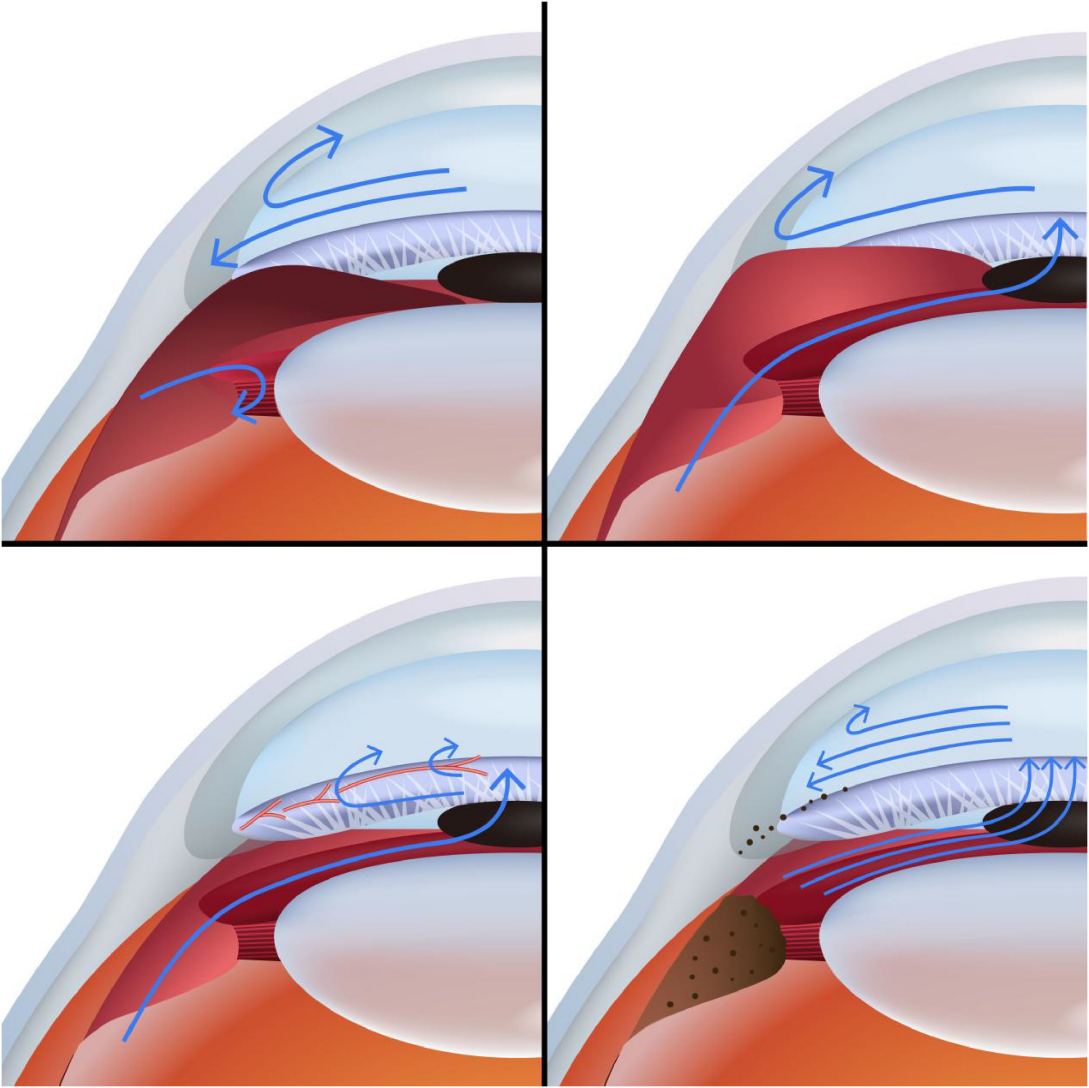
Common pathogenic mechanisms of tumor-related secondary glaucoma. The blue arrows in the figure simulate the production and drainage pathways of intraocular aqueous humor. Part A illustrates that under the influence of iris tumors, the posterior chamber space becomes narrow, the anterior chamber angle is narrowed, and the aqueous humor produced by the ciliary body is difficult to reach the anterior chamber due to pupillary block. At the same time, the fluid in the anterior chamber also struggles to get through the trabecular meshwork through the narrowed angle. Part B shows that secondary angle closure prevents aqueous humor from being drained through the trabecular meshwork. Part C illustrates the neovascular changes in the iris and anterior chamber angle, with the disease progression depicted in this static image encompassing three distinct phases: In the first phase, new vessels emerge on the trabecular meshwork without elevated IOP; in the second phase, fibrous tissue surrounds these vessels, impairing filtration and increasing IOP; in the third phase, fibrovascular contraction causes irreversible angle closure. Responses to anti-VEGF therapy vary across these stages. Part D illustrates that the ciliary body, stimulated by inflammatory factors, leads to increased concentrations of inflammatory factors and proteins in the aqueous humor (indicated by brown dots), resulting in blockage and fibrosis of the trabecular meshwork.

The pathogenesis of cancer-related glaucoma is complex and multifactorial, involving several key mechanisms. These include mechanical obstruction by tumor masses, secondary angle closure induced by various tumor-related factors, neovascularization driven by angiogenic mediators, and alterations in aqueous humor dynamics caused by tumor infiltration or debris. The essential features of these mechanisms are visually depicted in Figure 1.

#### 2.4.1 Mechanical obstruction

Mechanical obstruction is a key pathogenic mechanism. Tumors (such as iris/ciliary body tumors, melanoma cells, metastatic lesions) can impede aqueous humor outflow through three pathways:

I. Anatomical compression: as the tumor grows in size, it can push the root of the iris, narrowing or even closing the anterior chamber angle, and directly blocking the traditional aqueous humor outflow pathway composed of the trabecular meshwork (TM) - Schlemm’s canal (SC) (12, 13). For example, iris melanoma can cause pupillary block, increasing the resistance to aqueous humor outflow (12).
II. Tissue fibrosis: factors such as TGF-β2 released by tumors can promote the fibrosis of the TM, further increasing the outflow resistance (14).
III. Kinetic changes: mechanical compression can alter the biomechanical properties of the angle tissues, reducing the viscoelasticity of the TM and the wall of Schlemm’s canal, leading to disordered aqueous humor flow (15). Studies have shown that tumor mass can cause abnormal stress distribution in the TM/JCT region and can reduce the aqueous humor flow rate by up to 9% (16).

#### 2.4.2 Secondary angle closure

Tumor-related exudation (such as serous retinal detachment in uveal melanoma) or vitreous hemorrhage can cause posterior synechiae of the iris through mechanical obstruction or inflammatory reactions, leading to pupillary block and acute angle closure (17, 18). Studies show that approximately 7.47% of secondary glaucoma cases are associated with such complications (19). In clinical reports, patients developed refractory angle closure one month after being diagnosed with metastatic lung adenocarcinoma, which was identified to occur through choroidal metastasis. Traditional drug and laser treatments were ineffective, and the condition was finally relieved by radiotherapy (17). In addition, radiation can also damage the microvasculature of the TM, resulting in obstruction of aqueous humor outflow (20). It is worth noting that in patients with chronic primary angle-closure glaucoma (CPACG) complicated by radiation-induced maculopathy, the risk of ciliary body edema is significantly increased (21, 22).

#### 2.4.3 Neovascularization

Retinoblastoma and choroidal melanoma secrete angiogenic factors such as VEGF, which can directly stimulate the formation of new blood vessels in the iris and angle. At the same time, it activates the hypoxia-inducible factor (HIF-1α) signaling pathway, jointly promoting pathological angiogenesis (4, 23, 24). The regulation of HIF-1α further aggravates the microcirculation disorder of the retina, forming a “hypoxia-angiogenesis” vicious cycle (25–27). These neovascular changes progress through three distinct phases. In the first phase, new blood vessels begin to grow on the trabecular meshwork, yet IOP remains unchanged; anti-VEGF injections at this stage may prevent the development of NVG and subsequent IOP elevation. In the second phase, these new vessels become surrounded by fibrous tissue that covers the trabecular meshwork, impairing aqueous humor filtration and leading to increased IOP, where anti-VEGF injections combined with IOP-lowering medications may still help control IOP. In the third phase, the fibrovascular tissue contracts, pulling the iris forward and causing irreversible closure of the anterior chamber angle, such that anti-VEGF injections primarily serve to reduce the risk of bleeding during tube implant surgery. Ultimately, these new blood vessels can block the aqueous humor outflow channels, leading to increased intraocular pressure and NVG (23, 28, 29). Studies have shown that dual-target inhibition of VEGF/HIF-1α is more effective in reducing the neovascular area than single-target intervention (30), and anti-VEGF drugs have been proven to treat NVG by blocking this pathway (24). Retinal ischemia (such as in diabetic retinopathy and retinal vein occlusion) is the most common cause of NVG (28, 31).

#### 2.4.4 Inflammation and cytokine release

Tumor-associated inflammatory mediators disrupt ocular homeostasis through multiple pathways. For instance, in the case of blood-aqueous barrier disruption, IL-6 and TNF-α can upregulate vascular permeability, resulting in protein exudation, such as the deposition of IgG and complement C3. This also activates the macrophage-mediated inflammatory cascade (32–34). These inflammatory factors can further induce the expression of endothelial cell adhesion molecules, such as ICAM - 1/VCAM - 1, exacerbating the barrier dysfunction (35). Meanwhile, the exuded proteins and inflammatory mediators (such as IL-1β and MCP-1) co-deposit in the TM. They promote the abnormal accumulation of extracellular matrix (ECM) via the TLR4 signaling pathway, increasing the resistance to aqueous humor outflow (36, 37). Additionally, infections like HTLV - 1, a causative factor for leukemia/lymphoma, can cause TM cells to secrete excessive TNF-α and IL-6, directly impairing their contractile function (38, 39). In paraneoplastic syndromes, autoantibodies (such as anti-dsDNA antibodies) may target the surface antigens of TM cells, triggering complement activation and local inflammation (32, 40). Such autoimmune responses can act synergistically with pro-inflammatory factors released by tumors (such as IL-17 and TGF-β), accelerating the fibrosis of the TM (41).

#### 2.4.5 Alterations in aqueous humor dynamics

Tumor cells or necrotic debris blocking the TM spaces (such as intraocular infiltration in leukemia) can lead to secondary glaucoma by mechanically obstructing the aqueous humor outflow channels. Studies have shown that after tumor cells invade the TM spaces, they directly impede the outflow of aqueous humor, and the unphagocytosed melanin granules of pigmented tumors, such a melanocytoma, further exacerbate the obstruction (42).

Ciliary body tumors can cause elevated intraocular pressure through two mechanisms: First, when the ciliary body is compressed or infiltrated by a tumor, mechanical stretching stimuli may activate the Transient Receptor Potential Vanilloid 4-hemichannel mechanism in non-pigmented ciliary epithelial (NPE) cells. If the mechanical stimulation persists, it may cause aqueous humor secretion disorders through this mechanism. It should be noted that this also depends on the regulatory direction of signaling molecules such as Adenosine Triphosphate (ATP) (43). Second, the mass effect of the tumor alters the anatomical structure of the anterior chamber, leading to impaired aqueous humor drainage. Clinical observations have shown that for glaucoma associated with active malignant tumors, filtration surgery should be avoided, and giving priority to treating the primary tumor may normalize the intraocular pressure (44, 45).

#### 2.4.6 Secondary hemosiderosis

Secondary hemosiderosis is a pathological process in which hemosiderin abnormally deposits in ocular tissues such as the TM after long-term intraocular hemorrhage (such as retinal hemangioma or traumatic hemorrhage). Iron ions generate reactive oxygen species through the Fenton reaction, triggering oxidative stress and lipid peroxidation. This leads to dysfunction of TM cells and increased resistance to aqueous humor outflow, ultimately developing into open-angle glaucoma (46, 47). The clinical manifestations include heterochromia iridis, brownish - black deposition in the TM, and elevated intraocular pressure (48). Histopathological examination shows that iron deposition directly damages retinal ganglion cells, accelerating glaucomatous optic neuropathy (46, 49).

Table 1 summarizes the key pathogenesis mechanisms, clinical manifestations, and associated references across tumor types causing cancer-related glaucoma, facilitating a clear overview of their distinct features and links.

**TABLE 1:** Pathogenesis, clinical manifestations, and prevalence of cancer-related glaucoma by tumor type.

Tumor type	Pathogenesis mechanisms	Clinical manifestations	Prevalence/risk factors	References
Ocular tumors (iris melanoma, retinoblastoma)	1. Mechanical obstruction: Tumor growth compresses iris root, narrows anterior chamber angle, and causes pupillary block; tumor-released TGF-β2 promotes trabecular meshwork (TM) fibrosis. 2. Neovascularization: Tumor-secreted VEGF activates HIF-1α signaling pathway, inducing neovascularization in iris and anterior chamber angle. 3. Inflammation: Tumor-associated IL-6, TNF-α disrupt blood-aqueous barrier, leading to protein exudation and TM fibrosis.	1. Rapid elevation of intraocular pressure (IOP), corneal edema, and sharp vision decline. 2. Prominent neovascularization on iris surface, often extending radially toward the periphery; fibrovascular membranes in anterior chamber angle. 3. Iris tumors (86% pigmented, 14% amelanotic); hyphema and vitreous seeding in retinoblastoma, with “pseudo-hypopyon” due to tumor cell deposition.	1.22% of uveal melanoma cases develop secondary glaucoma due to neovascularization; 76% due to angle narrowing or closure. 2.43% of eyes with iris melanoma and elevated IOP have ciliary body involvement, a significantly higher prevalence than eyes without elevated IOP (21%).	(24, 27, 50–52)
Metastatic tumors (lung adenocarcinoma, breast cancer)	1. Secondary angle closure: Choroidal metastasis causes anterior chamber shallowing; radiotherapy induces ciliary body edema and choroidal thickening, compressing angle structures. 2. Mechanical obstruction: Tumor infiltration of anterior chamber angle blocks aqueous humor outflow.	1. Angle closure accompanied by elevated intraocular pressure. 2. Choroidal thickening and serous retinal detachment; “vascular void” phenomenon detected by OCTA.	1.Over 90% of adult intraocular tumors are metastatic, with 75-85% originating from lung or breast cancer; 88% involve choroid. 2.35% of cases develop secondary IOP elevation, with higher incidence in breast cancer metastases (41%) than lung cancer metastases (29%).	(17, 53–55)
Intracranial tumors (meningiomas)	Elevated cerebrospinal fluid pressure may cause optic nerve edema and then atrophy, which can be confused with damage from glaucoma, even without elevated IOP.	1. Asymmetric visual field defects; optic disk edema in acute cases, nerve fiber layer thinning in chronic cases. 2. May mimic primary open-angle glaucoma, requiring differential diagnosis.	Among the etiologies of optic disc edema, intracranial hypertension related to intracranial lesions accounts for 52%, which is significantly higher than other causes such as tumor infiltration (17%) and optic nerve compression (12%).	(56, 57)
Hematologic malignancies (leukemia)	1. Alterations in aqueous humor dynamics: Tumor cells or necrotic debris block TM spaces. 2. Secondary hemosiderosis: Long-term intraocular hemorrhage leads to hemosiderin deposition in TM, inducing oxidative stress and TM dysfunction.	1. TM obstruction by tumor cells or melanin granules. 2. Heterochromia iridis; brownish-black deposition in TM; elevated IOP.	Tumor cell infiltration of TM directly impedes aqueous humor outflow; unphagocytosed melanin granules exacerbate obstruction.	(42, 45, 48)
Pediatric tumors (optic pathway gliomas, retinoblastoma)	1. Neovascularization: Retinal ischemia drives neovascular glaucoma (NVG). 2. Mechanical obstruction: Tumor mass effect narrows anterior chamber angle. 3. Gliomas cause atrophy due to compression of the optic nerve	1. Leukocoria in retinoblastoma. 2. Refractory glaucoma in 22% of retinoblastoma cases due to angle infiltration or iris neovascularization. 3. Up to 54% of optic pathway gliomas develop secondary optic nerve atrophy.	Secondary glaucoma accounts for 53.4% of pediatric glaucoma cases; 28% are associated with acquired systemic diseases. Some are from systemic or ocular tumors.	(5, 6, 52, 58)

TGF-β2, transforming growth factor-beta 2; VEGF, vascular endothelial growth factor; HIF-1α, hypoxia-inducible factor-1 alpha; IL-6, interleukin-6; TNF-α, tumor necrosis factor-alpha; IOP, intraocular pressure; TM, trabecular meshwork; NVG, neovascular glaucoma; OCTA, optical coherence tomography angiography.

## 3 Clinical manifestation

### 3.1 Rapid increase in intraocular pressure

Glaucoma secondary to tumors can present as acute angle-closure glaucoma, characterized by sudden onset of headache, nausea, corneal edema, and a sharp decline in vision. Iris tumors, such as melanomas, can cause acute intraocular pressure elevation through pupillary block or direct invasion of the anterior chamber angle. Among patients with uveal melanoma, 22% develop secondary glaucoma due to neovascularization in the anterior chamber, while 76% have it due to angle narrowing or closure, and some of these patients have direct tumor invasion of the anterior chamber angle (50).

### 3.2 Neovascular changes

Prominent neovascularization on the iris surface, often extending radially toward the iris periphery, and fibrovascular membranes in the anterior chamber angle are typical manifestations of NVG, commonly encountered in tumor-related ischemia, including retinoblastoma and metastatic tumors (4). Approximately 71% of cases of glaucoma secondary to uveal melanoma have neovascularization as the main mechanism (50).

### 3.3 Tumor-related characteristic signs

I. Iris tumors: iris nodules should be studied with UBM to differentiate cystic from solid lesions. most solid lesions are melanoma, which is the most common primary uveal malignancy. Iris melanoma can present as a localized iris elevation or a space-occupying lesion of the ciliary body. clinical studies have found that approximately 43% of cases are associated with ciliary body involvement, which is significantly higher than that in patients without glaucoma (21%) (50). Iris tumors are usually pigmented (about 86%), but 14% of patients present with an amelanotic variant (51).
II. Hyphema: children with retinoblastoma may develop hyphema and vitreous seeding, accompanied by the characteristic leukocoria (2, 59). This phenomenon is caused by blood vessel rupture due to tumor necrosis or bleeding from new blood vessels. multimodal imaging shows that the hyphema is often accompanied by the deposition of tumor cells on the surface of the iris, resembling “pseudo-hypopyon” (52). It is worth noting that approximately 22% of cases develop refractory glaucoma secondary to tumor cell blockage of the anterior chamber angle or iris neovascularization. Such patients often require urgent treatment to preserve the eyeball (60). Differential diagnosis should include Coats’ disease, persistent hyperplastic primary vitreous (PHPV), etc., (61).
III. Choroidal thickening/detachment: in metastatic intraocular tumors (accounting for more than 90% of adult intraocular tumors), breast cancer and lung cancer metastases account for 75%–85% (53). these metastases most commonly involve the choroid (88%), presenting as rapidly progressive choroidal thickening (with an average thickness of 2.3 ± 0.7 mm) and serous retinal detachment (54). optical coherence tomography angiography (OCTA) can detect the characteristic “vascular void” phenomenon, which is in contrast to the “vascular arcade” structure of primary choroidal melanoma (62). approximately 35% of cases develop secondary elevation of intraocular pressure due to mechanical compression or inflammatory reactions. the incidence is significantly higher in breast cancer metastases (41%) than in lung cancer metastases (29%) (55).

### 3.4 Visual field and optic nerve damage

Tumor compression or ischemia can lead to early asymmetric visual field defects. Intracranial hypertension secondary to tumors is a common cause of optic disk edema (56). Acute angle-closure glaucoma may be accompanied by optic disk edema, while chronic cases show thinning of the nerve fiber layer (57, 63). Among patients with glaucoma secondary to uveal melanoma, 53% have retinal detachment, which is significantly higher than that in the non-glaucoma group (30%) (50).

### 3.5 Involvement of the contralateral eye

Involvement of the contralateral eye is a prominent feature of paraneoplastic ophthalmic syndromes, and it is particularly evident in cancer-associated retinopathy (CAR) and anti-Hu antibody-related optic neuritis (64). In CAR, both retinas are often affected simultaneously, presenting with visual acuity decline, visual field defects, and abnormal electroretinogram, which is caused by photoreceptor dysfunction. Approximately 60%–70% of CAR cases are associated with small-cell lung cancer (SCLC), and the anti-Hu antibody is a common paraneoplastic antibody in SCLC (65, 66). Anti-Hu antibody-positive optic neuritis leads to rapid bilateral visual loss, often accompanied by eye pain and pupillary afferent defects. Approximately 80% of patients are diagnosed with SCLC, and optic neuritis may precede the diagnosis of the tumor by several months (67, 68). MRI can show optic nerve enhancement, and a negative cerebrospinal fluid antibody test does not rule out a paraneoplastic etiology (69). When the intraocular pressure is elevated, it needs to be differentiated from primary glaucoma (70, 71).

### 3.6 Systemic associated symptoms

Approximately 11% of patients with uveal melanoma and newly diagnosed secondary glaucoma have synchronous metastases, which is significantly higher than 1.2% in the non-glaucoma group (50). In some cases, ocular symptoms precede the diagnosis of the primary tumor. For example, breast cancer metastases may present as vitreous opacities or choroidal masses (2, 72).

## 4 Treatment strategies

### 4.1 Supplementary information on primary tumor treatment

I. In terms of targeted therapy, the combined use of FGFR4 and EZH2 inhibitors offers a new strategy for hepatic cell carcinoma with eye metastases (73). Similar approaches may be extended to primary ocular tumors. Besides controlling neovascularization, anti-VEGF drugs, when combined with gene therapy, have shown a synergistic effect in intraocular malignancies (54, 74).
II. For uveal melanoma, although traditional radiotherapy and surgery remain the mainstream, inhibitors targeting ERRs (estrogen-related receptors) may be a breakthrough point by reversing tumor drug resistance (75). The latest clinical data show that immune checkpoint inhibitors have an objective response rate of 35%–40% for conjunctival melanoma and squamous cell carcinoma (76).

### 4.2 Surgical innovations and contraindications

I. For tumors with orbital invasion, modified orbital exenteration combined with intraoperative radiotherapy can increase the negative margin rate (60). As this procedure removes the eye and orbital contents, it inherently eliminates secondary glaucoma in the affected eye, rendering additional glaucoma surgery unnecessary. For tumors without orbital invasion and requiring glaucoma management, filtration surgery alternatives such as glaucoma drainage valve implantation should be preceded by ultrasound biomicroscopy (UBM) to confirm the absence of anterior chamber tumor invasion (77). Notably, invasive procedures like intravitreal injections, trabeculectomies, or tube-shunts in eyes with primary or metastatic tumors carry risks of tumor seeding, spread, or exacerbating inflammation; these are relatively contraindicated unless strictly necessary, with priority given to controlling the primary tumor while using conservative IOP-lowering strategies. However, anti-VEGF agents may be administered systemically in such cases, in close collaboration with the treating oncologist, to balance anti-angiogenic effects and tumor management.
II. Eye drops to reduce intraocular pressure, combined with intraocular anti-VEGF agent injections or glucocorticoid injections, based on the specific mechanisms of secondary glaucoma, may be considered (78, 79). Additionally, ultrasound cycloplasty, an emerging non-invasive procedure, or other cylodestructive/cyclomodulation procedures, can be employed when necessary (80).
III. Local excision of eyelid tumors combined with intraoperative cryotherapy can achieve a success rate of 82% in small tumors (< 8mm) while preserving the eyeball (81).

### 4.3 New progress in multidisciplinary collaboration

I. Remote consultation systems (such as EE - Explorer) can accelerate the initial diagnosis of emergency ocular tumors, especially applicable to cases like retinoblastoma that require urgent intervention (82, 83).
II. Pediatric neuro-ocular tumors require joint assessment by neuro-ophthalmology, as 54% of cases with optic pathway gliomas develop secondary optic nerve atrophy (58).
III. Special precautions: α2 adrenergic agonists (such as brimonidine) can be used as an alternative to prostaglandin analogs in patients with uveal melanoma (UM), and they may be safer (84). Studies have shown that α2 agonists have anti-angiogenic effects (85) and can modulate the tumor microenvironment (86). In contrast, prostaglandin analogs are significantly associated with an increased risk of UM metastasis (87). Additionally, α2-agonists have a comparable effect on intraocular pressure control to other drugs (88). However, it should be noted that there may be neurocognitive risks associated with long-term use (89).
IV. Future directions: ferroptosis-based nanophotosensitizers (such as Mn-doped porphyrin frameworks) have shown precise killing effects on intraocular tumors in animal models (90, 91).
V. Artificial intelligence-assisted pathological diagnosis systems are optimizing the decision-making of targeted therapy for ocular tumors (92).

## 5 Future prospects

### 5.1 Tailor strategies to individuals

The therapeutic prospects of tumor-related secondary glaucoma require strategy formulation based on etiologies and risk factors. Studies have shown that the main mechanisms of glaucoma secondary to uveal melanoma are anterior chamber angle narrowing (76% at diagnosis) and neovascularization (71% during follow-up) (50). For high-risk populations, enhanced monitoring of intraocular pressure and neovascularization is necessary. Future research may explore preventive anti-vascular endothelial growth factor injections while optimizing radiotherapy regimens to reduce complications such as angle synechia. Treatment requires multidisciplinary collaboration, integrating tumor control with glaucoma management to balance vision protection and quality of life (93).

### 5.2 Gene therapy

The prospects of gene therapy for tumor-related secondary glaucoma primarily lie in intervening in the disease process by targeting the regulation of genes related to intraocular pressure or providing neuroprotective effects on retinal ganglion cells. Adeno-associated virus vectors have become a commonly used delivery tool due to their high targeting and low immunogenicity, with preclinical studies showing their ability to reduce intraocular pressure and delay optic nerve degeneration (94, 95). Additionally, the combination of nanomedicine and gene therapy, such as stem cell membrane-coated nanoparticles, can improve drug delivery efficiency, offering new strategies for regulating the tumor microenvironment (96, 97). Although the existing research does not directly focus on therapeutic studies of tumor-related secondary glaucoma, it provides valuable insights into the therapeutic prospects of this condition.

### 5.3 Integration of multimodal imaging

Optical coherence tomography (OCT) angiography, can be combined with fluorescein angiography to improve the detection rate of neovascularization in the anterior chamber angle, but the limitation of the resolution of deep vessel imaging needs to be addressed (98). Combining liquid biopsy (such as the detection of exosomes in aqueous humor) may enhance the specificity of early diagnosis (50, 99).

### 5.4 Optimization of AI models

Current deep learning models are mostly based on single-modal data (98). In the future, clinical parameters (such as the intraocular pressure fluctuation curve and tumor volume) and omics data (such as mitochondrial DNA, mtDNA mutations) need to be incorporated to construct a multi-dimensional prediction system (100, 101), and its generalization ability needs to be verified through prospective cohorts.

## 6 Conclusion

Glaucoma secondary to tumors is a severe complication triggered by primary or metastatic tumors and their treatments, with complex and diverse pathogenic mechanisms. Mechanical obstruction (such as tumor compression of the anterior chamber angle), neovascularization, fibrosis of the TM mediated by inflammatory factors (IL-6, TNF-α), abnormal aqueous humor dynamics, and angle closure induced by either pupillary block or iris/cilliary body invasion are the main pathological mechanisms. The clinical manifestations are heterogeneous, including acute elevation of intraocular pressure, iris neovascularization, choroidal thickening, and characteristic tumor signs. Treatment requires multidisciplinary collaboration, with the control of the primary tumor as the core, combined with anti-VEGF drugs, targeted therapies (such as FGFR4/EZH2 inhibitors), and modified surgeries (such as cryotherapy combined with local excision), while avoiding the use of filtration surgery in active tumors. Future research will focus on personalized therapeutic strategies, gene therapy applications, integration of multimodal imaging, and optimization of AI models, to improve early intervention strategies and reduce the risk of irreversible optic nerve damage.
